# Transverse Magnetic Surface Plasmons in Graphene Nanoribbon Qubits: The Influence of a VO_2_ Substrate

**DOI:** 10.3390/nano13040718

**Published:** 2023-02-13

**Authors:** Mousa Bahrami, Panagiotis Vasilopoulos

**Affiliations:** 1 Bita Quantum AI Inc., 2021 Av. Atwater, Montréal, QC H3H 2P2, Canada; 2 Department of Physics, Concordia University, 7141 Sherbrooke Ouest, Montréal, QC H4B 1R6, Canada

**Keywords:** surface plasmon, graphene nanoribbon, Lindhard approximation, quantum wire, VO_2_, phase-change materials, substrate-induced band gap, qubit

## Abstract

We study the influence of the phase-change material VO_2_ on transverse magnetic (TM) surface plasmon (SP) modes in metallic arm-chair graphene nanoribbon (AGNR) qubits in the Lindhard approximation. We assess the effects of temperature as a dynamic knob for the transition from the insulating to the metallic phase on the TM SP modes in single-band (SB) and two-band (TB) transitions. We show that a VO_2_ substrate leads to TM SP modes in both SB and TB transitions. In addition, we observe that the SP modes have a lower frequency than those for a substrate of constant permittivity. In addition, we study the influence of the substrate-induced band gap $\Delta^{\prime }$ on SP modes in TB transitions for the insulating and metallic phases of VO_2_.

## 1. Introduction

Analyzing and processing big data is one of the biggest challenges of the third millennium [1,2]. These data can be found in many areas of science, technology, and industry, such as weather forecasting and climate change, logistic optimization, financial modeling, space colonization, the pharmaceutical industry, artificial intelligence, cybersecurity, space exploration, etc. [3,4,5,6,7,8,9,10]. Nowadays, we exploit semiconductor technology to process this ocean of information when developing and designing computational units, such as CPUs. The clock speeds of these CPUs, due to fundamental limitations imposed by quantum mechanics and the laws of thermodynamics and electrodynamics, are limited to only a few GHz [11,12]. Employing multi-core CPU architectures and parallel programming methods has been one of the brilliant approaches to overcoming this challenge and achieving the fast processing of information [13]. However, more is needed to analyze data in real time.

One of the best candidates for addressing this challenge is harnessing the power of quantum computers with their unprecedented speed. For instance, a complex problem such as quantum supremacy needs hundreds of thousands of classical computers to run for ten thousand years, while with a powerful quantum computer, this time is reduced to a few minutes [14,15]. Today’s most noisy intermediate-scale quantum computers, which are built by companies such as IBM, Google, and Microsoft, utilize superconductor qubits as quantum processors. Superconductor qubits operate at very low temperatures—close to zero Kelvin. This restricts their scalability and integration with photonic technology. In addition, the clock speeds of these qubits reach up to a few GHz, and error correction [16,17,18] is an obstacle that has not been fully addressed in this technology so far.

One of the solutions for overcoming all of these challenges is the use of plasmonic waves in quantum materials whose thickness is about one atom, such as graphene [19,20,21,22,23]. In this case, the qubits (i) operate at room temperature, (ii) their clock speed is about a few THz, and (iii) they have no need for error mitigation; in addition, they can be easily integrated with optical fibers [24]. In many studies, changing the Fermi energy was considered the only dynamic approach to controlling the spectrum of plasmon waves in graphene qubits [25,26,27,28,29,30,31,32,33]. These studies assumed that the permittivity or temperature of the substrate is constant. However, the emergence of phase-change materials (PCMs), such as VO_2_, which can transition from an insulating to a metallic phase by varying the temperature, has opened a new horizon for dynamically controlling the state of graphene qubits [34,35,36,37,38,39]. In this paper, we study the influence of a VO_2_ substrate phase change with temperature on the TM surface plasmon modes of arm-chair graphene nanoribbons as qubits for single-band (SB) and two-band (TB) transitions, which are also known as intra-band and inter-band transitions. Note that, in our previous paper, we studied the influence of VO_2_ on plasmon modes in AGNRs [40]. It is worth pointing out that plasmon modes are longitudinal electromagnetic fields propagating inside AGNRs, while surface plasmon modes are TM or TE evanescent electromagnetic fields propagating at the interface of AGNRs with the surrounding media. It should be noted, though, that metallic AGNRs do not support TE SP modes because there is no conductivity along the width of the ribbons.

**Figure 1:**
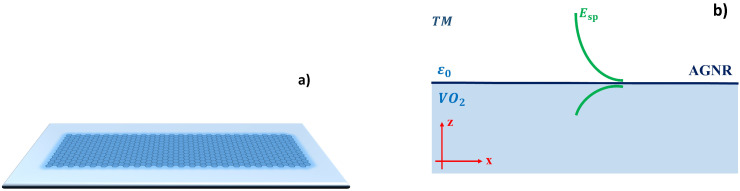
(**a**) An AGNR grown on a VO_2_ substrate. (**b**) Propagation of a TM SP mode along the interface of VO_2_ and air in the presence of an AGNR.

In this work, we study TM SP modes in an AGNR on a VO_2_ substrate, as sketched in Figure 1, without considering many-body effects, such as electron–electron interaction and exchange. This paper is organized as follows. In Section 2, we present the general expression for TM SP modes within the Lindhard approximation by considering the influence of the energy gap induced by the substrate. Section 3 discusses the results the obtained in the presence and absence of VO_2_ for SB and TB transitions. Our summary follows in Section 4.

## 2. Formalism

The TM SP modes of a quantum material that is one atom thick and sandwiched between two media can be obtained by finding the roots of the real part of [41,42,43]

(1) $$q\sigma \left(q,\omega \right)=i\omega \left[\epsilon_{1}\left(q,\omega \right)+\epsilon_{2}\left(q,\omega \right)\right],$$

where $\epsilon_{1}$ and $\epsilon_{2}$ are the permittivities of the surrounding media. Note that *q* and ω denote the wave vector and frequency of the excited surface plasmon mode. In Equation (1), σ denotes the conductivity of the quantum material given by [44]:

(2) $$\sigma \left(q,\omega \right)=ie^{2}\omega \chi \left(q,\omega \right)/q^{2},$$

with *e* and χ being the electron charge and polarization function, respectively [45]. In the absence of many-body effects, such as Coulomb interaction or exchange effects, this quantity for AGNRs and SB transitions is given by [40]:

(3) $$\begin{array}{c} \chi^{SB}\left(q^{\prime },\omega^{\prime }\right)=\frac{1}{2W\pi \hbar v_{F}^{*}}\;\\ \times \left\{\begin{array}{ccc} \frac{2q^{\prime 2}}{\omega^{\prime 2}-q^{\prime 2}}+i\pi q^{\prime }\left[\delta \left(q^{\prime }+\omega^{\prime }\right)-\delta \left(q^{\prime }-\omega^{\prime }\right)\right],\; & \; & q\leq k_{F},\;\\ \frac{2q^{\prime }}{\omega^{\prime 2}-q^{\prime 2}}+\frac{1}{2}Ln\left[\frac{{\left(2+\omega^{\prime }\right)}^{2}-q^{\prime 2}}{\omega^{\prime 2}-q^{\prime 2}}\right]+i\pi \left[\delta \left(q^{\prime }+\omega^{\prime }\right)-\delta \left(-q^{\prime }+\omega^{\prime }\right)\right],\; & \; & q>k_{F}, \end{array}\right.\; \end{array}$$

and for TB transitions, it is given by:

(4) $$\begin{array}{c} \chi^{TB}\left(q^{\prime },\omega^{\prime }\right)=\frac{1}{2W\pi \hbar v_{F}^{*}}\;\\ \times \left\{\begin{array}{ccc} \frac{2q^{\prime }\left(q^{\prime }+\Delta^{\prime }\right)}{{\left(q^{\prime }+\Delta^{\prime }\right)}^{2}-\omega^{\prime 2}}+\frac{1}{2}Ln\left(\frac{{\left(3q^{\prime }+\Delta^{\prime }\right)}^{2}-\omega^{\prime 2}}{{\left(q^{\prime }+\Delta^{\prime }\right)}^{2}-\omega^{\prime 2}}\right)+i\pi q^{\prime }\left[\delta \left(q^{\prime }+\Delta^{\prime }-\omega^{\prime }\right)-\delta \left(q^{\prime }+\Delta^{\prime }+\omega^{\prime }\right)\right], & \; & q\leq k_{F},\;\\ \frac{2(q^{\prime }+\Delta^{\prime })}{{\left(q^{\prime }+\Delta^{\prime }\right)}^{2}-\omega^{\prime 2}}+\frac{1}{2}Ln\left(\frac{{\left(2+\Delta^{\prime }+q^{\prime }\right)}^{2}-\omega^{\prime 2}}{{\left(q^{\prime }+\Delta^{\prime }\right)}^{2}-\omega^{\prime 2}}\right)+i\pi \left[\delta \left(q^{\prime }+\Delta^{\prime }-\omega^{\prime }\right)-\delta \left(q^{\prime }+\Delta^{\prime }+\omega^{\prime }\right)\right], & \; & q>k_{F}, \end{array}\right.\; \end{array}$$

where $q^{\prime }=q/k_{F}$, $\omega^{\prime }=\hbar \omega /E_{F}$, and $v_{F}^{*}$ are the dimensionless wave vector, frequency, and renormalized Fermi velocity, respectively [46]. Note that $\Delta^{\prime }=\Delta /E_{F}$, where Δ indicates the energy gap induced by the substrate on the polarization function of AGNRs. *W* indicates the width of the ribbon given by $W=\sqrt{3}\left(N+1\right)a_{cc}/2$, where *N* and $a_{cc}$ denote the dimer number and distance between two carbon atoms, respectively [47,48,49,50,51,52].

## 3. Results and Discussion

### 3.1. Substrates with Constant Permittivity

**Figure 2:**
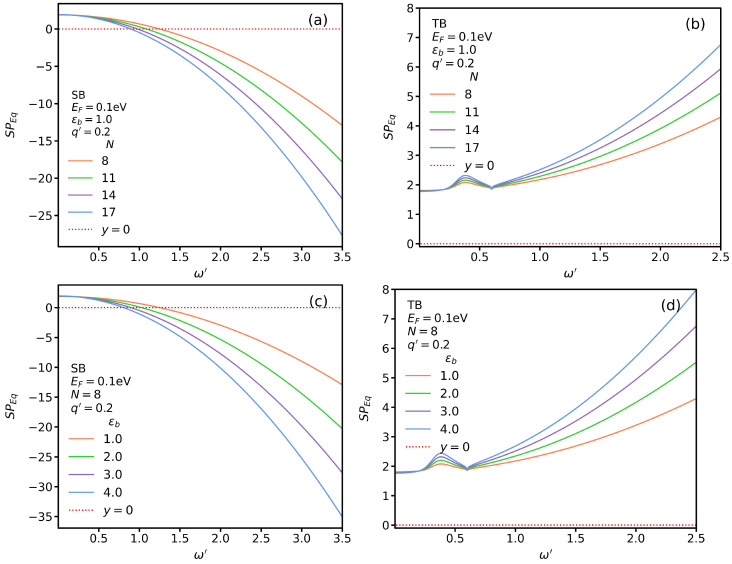
(**a**,**b**) SP*_Eq_* for SB and TB transitions, respectively, for several widths N∝W, with $E_{F}=0.1$ eV and $\epsilon_{b}=1$. (**c**,**d**) The same as in (**a**,**b**), respectively, for different values of $\epsilon_{b}$ and fixed N=8.

In Figure 2a,b, we plot Equation (1) for suspended AGNRs in which $\epsilon_{1}=\epsilon_{2}=\epsilon_{0}$ for several widths and a fixed momentum, $q^{\prime }=0.2$, for SB and TB transitions, respectively. The Fermi energy and renormalized Fermi velocity are $E_{F}=0.1$ eV and $10^{6}$ m/s. Notice that we use the following notation in our plots: $\epsilon_{b}=\epsilon_{2}/\epsilon_{0}$. The TM SP modes are the roots of Equation (1). In other words, as depicted, the root of the real part of Equation (1) is where a graph intersects with the dashed red line, $SP_{Eq}=0$, where we use the convention SPEq≡Re(qσ−iω(ϵ1+ϵ2)). While no TM SP modes exist for the TB transition, as shown in Figure 2b, we see that for SB transitions, the SP mode frequency decreases with increasing nanoribbon width *W*. In addition, to study the influence of the substrate on SP modes, we plot SP*_Eq_* for several different constants $\epsilon_{2}$ for SB and TB transitions in Figure 2c,d, respectively, for a fixed width of N=8. Similarly to Figure 2b, in this case, no SP modes exist for TB transitions. In Figure 2c, we observe that substrates with a higher permittivity result in SP modes with lower frequencies.

**Figure 3:**
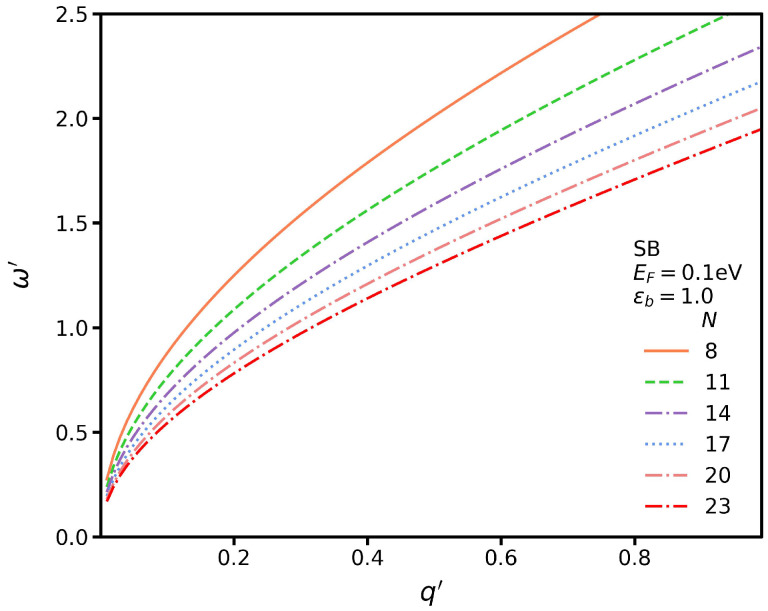
Dispersion of TM SP modes (SB transitions) for several different widths but fixed $\epsilon_{b}=1$.

In Figure 3, we plot the TM SP dispersion for several different *N*s. We see that for a fixed momentum, the SP energy decreases as the width increases. In addition, in this case, an analytic expression can be found for the SP dispersion, which reads

(5) $$\begin{array}{c} \omega^{\prime }=\sqrt{q^{\prime 2}+\zeta q^{\prime }}, \end{array}$$

where $\zeta =2e^{2}/\pi \sqrt{3}\left(\epsilon_{1}+\epsilon_{2}\right)\left(N+1\right)a_{cc}E_{F}$. The SP dispersion shows a general trend in which the plasmon frequency increases with the momentum. Additionally, higher momentum values produce SP modes with greater differences compared to those with a lower momentum.

### 3.2. Substrates with Phase-Change Functionality: VO_2_

To express the permittivity of temperature-dependent phase-change materials, such as VO_2_, we use the Maxwell–Garnet approximation given by [53]:

(6) $$\epsilon_{MG}=\epsilon_{ins}\frac{\epsilon_{met}\left(2F+1\right)+\epsilon_{ins}\left(2-2F\right)}{\epsilon_{met}\left(1-F\right)+\epsilon_{ins}\left(2+F\right)},$$

where *F* is the ratio between the metallic and insulating phases and is known as the filling factor. It is given by [54]:

(7) $$F\left(T\right)=\frac{1}{1+e^{2\left(T_{0}-T\right)/T_{c}}},$$

with $T_{0}$ = 70.5 °C and $T_{c}$ = 4 °C. Note that, in Equation (6), $\epsilon_{met}$ and $\epsilon_{ins}$ are the permittivities for the metallic and insulating phases, and they are, respectively, given by:

(8) $$\frac{\epsilon_{ins}\left(\omega \right)}{\epsilon_{0}}=1+\frac{a_{0}}{1-{\left(\omega /\omega_{0}\right)}^{2}}+\sum_{j=1}^{7}\frac{a_{j}}{1-{\left(\omega /\omega_{j}\right)}^{2}-ib_{j}\left(\omega /\omega_{j}\right)},$$

(9) $$\frac{\epsilon_{met}\left(\omega \right)}{\epsilon_{0}}=1+\frac{c_{0}}{1-{\left(\omega /\omega_{0}\right)}^{2}}+\frac{\omega_{p}^{2}}{\omega (\omega +i\gamma )}+\sum_{j=1}^{4}\frac{c_{j}}{1-{\left(\omega /\Omega_{j}\right)}^{2}-id_{j}\left(\omega /\Omega_{j}\right)},$$

with $a_{0}=3.26$, $c_{0}=2.95$, $\hbar \omega_{0}=15$ eV, $\hbar \omega_{p}=3.33$ eV, and ℏγ=0.66 eV. In addition, the $a_{j}$ and $c_{j}$ coefficients are provided in Table 1 and Table 2 [55].

**Figure 4:**
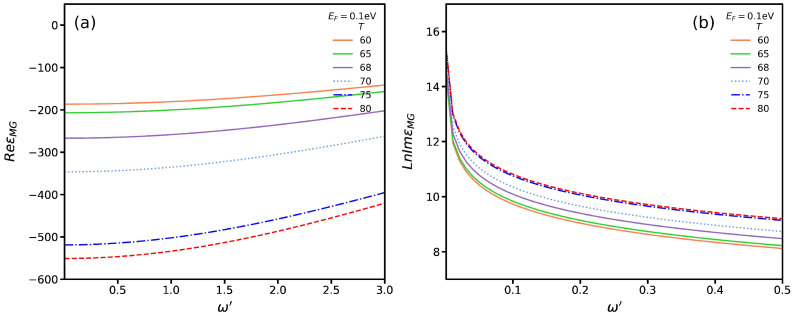
(**a**) Real and (**b**) imaginary parts of the Maxwell–Garnet permittivity of VO_2_ for several temperatures.

In Figure 4a,b, we plot, respectively, the real and imaginary parts of the permittivity of VO_2_ as functions of frequency for several temperatures. Notice that VO_2_ is in the insulating (F=0) and metallic (F=1) phases for *T* < 60 °C and *T* > 80 °C, respectively. Moreover, for 60<T<80, with 0<F<1, it is in a mixture of both phases. In Figure 4a, we see a general trend in which increasing the frequency causes the value of the real part of $\epsilon_{MG}$ to increase. In addition, we observe that the magnitude of this quantity in the insulating phase is larger than that of the metallic phase. We can say that this magnitude is about two times larger in the insulating phase than that in the metallic one. In Figure 4b, we can also see a general trend in which, for lower frequencies, the magnitude of the imaginary part of $\epsilon_{MG}$ dramatically increases. However, the value of this quantity for the metallic phase is larger than that in the insulating one. In other words, the energy dissipation in the metallic phase is larger than that in the insulating phase. It is worth noting that, for lower frequencies, the behavior of $\epsilon_{MG}$ resembles that of the Drude model, as demonstrated in part (b) of Figure 4. It is reasonable to expect this outcome, as the terms ${\left(\omega /\omega_{j}\right)}^{2}$ and ${\left(\omega /\Omega_{j}\right)}^{2}$ in the sum of (8) and (9) can be disregarded for low frequencies.

**Figure 5:**
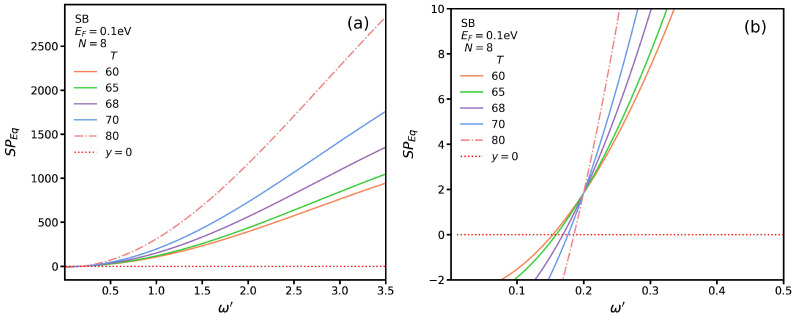
SP*_Eq_* for SB transitions as a function of $\omega^{\prime }$ at several temperatures for (**a**) $\omega^{\prime }<3.0$ and (**b**) $\omega^{\prime }<0.5$.

In Figure 5a, we display the graph of SP*_Eq_* for SB transitions at different temperatures as a function of dimensionless frequency for a fixed momentum of $q^{\prime }=0.2$, a Fermi energy of $E_{F}=0.1$ eV, and a width of N=8. To find the roots of the equation, we plotted it for $\omega^{\prime }\leq 0.5$ and identified the intersections of the graph with the dotted red line in Figure 5b. We observe that at the intersection point, the SP mode frequency increases with the temperature. In other words, the frequency of the SP modes in the insulating phase is smaller than that in the metallic one.

**Figure 6:**
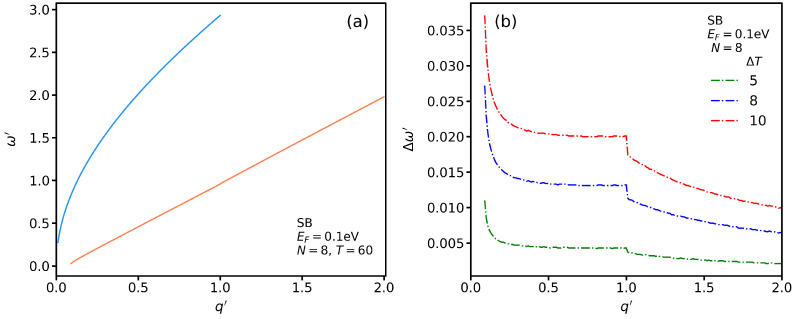
(**a**) SB SP modes in the presence (red curve) and absence (blue curve) of VO_2_ for N=8 and $E_{F}=0.1$ eV. (**b**) $\Delta \omega^{\prime }$ SP modes in the presence of VO_2_ for several different values of ΔT.

In Figure 6a, we plot the SB SP mode dispersion for N=8 and $E_{F}=0.1$ eV. The blue graph indicates the suspended AGNRs, $\epsilon_{1}=\epsilon_{2}=\epsilon_{0}$, which are denoted by *bar*, while the orange graph denotes the SP spectrum for the insulating phase of VO_2_ (*T* = 60 °C). In contrast to the *bar* spectrum, which is limited for this width (N=8 to $q^{\prime }\leq 1$), the SP modes in the presence of VO_2_ extend beyond $q^{\prime }=1$. In other words, the influence of VO_2_ as a phase-change material results in SP modes with higher wave vectors and lower frequencies. To show the difference between the SB SP plasmon modes for different temperatures, we set $\Delta \omega^{\prime }$ as the difference between the plasmon frequency at temperatures *T* and *T* = 60 °C in Figure 6b. We see a general trend in which, by increasing $q^{\prime }$, the magnitude of $\Delta \omega^{\prime }$ decreases and then becomes constant up to a point that we call the critical wave vector, $q_{c}$. Beyond $q_{c}^{\prime }$, $\Delta \omega^{\prime }$ decreases more gently. We observe that $\Delta \omega^{\prime }$ increases when ΔT is increased for a fixed $q^{\prime }$. That is, the energy of SP modes increases by changing from the insulating to the metallic phase.

**Figure 7:**
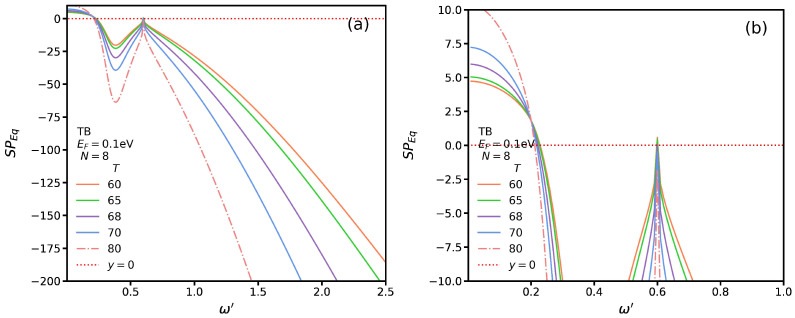
SP*_Eq_* for TB transitions as function of $\omega^{\prime }$ at several temperatures for (**a**) $\omega^{\prime }<2.5$ and (**b**) $\omega^{\prime }<1$.

In Figure 7a, we plot SP*_Eq_* for TB transitions and several temperatures with $\Delta^{\prime }=0$ and a typical momentum such that $q^{\prime }=0.2$. To more clearly see its roots—the intersections of the curves with the dotted line—we replot it in Figure 7b for $\omega^{\prime }\leq 1$. While there is only one root for the SP mode and SB transitions, we observe three SP modes for the TB transitions. The first root of the metallic phase occurs at a lower frequency than that in the insulating phase. It seems that the other two SP modes with higher frequencies for each temperature converge to similar values.

**Figure 8:**
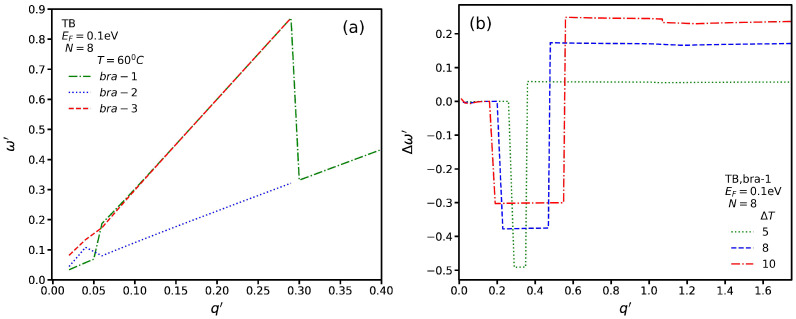
(**a**) Three SP-mode spectra for TB transitions at *T* = 60 °C. (**b**) $\Delta \omega^{\prime }$ for several ΔT.

However, if we plot the SP spectrum, as shown in Figure 8a for the insulating phase, we see that, in some range of $q^{\prime }$, a degeneracy exists for SP modes. We use the convention *bar-1*, *bar-2*, and *bar-3* to distinguish the three SP spectrum branches. We observe that for $q^{\prime }\leq 0.05$, there is no degeneracy for SP modes. While *bar-1* exists for all wave vectors, we notice that the *bar-2* and *bar-3* branches exist only for $q^{\prime }\leq 0.3$. Although *bar-3* acts as a monotonic function with a constant group velocity, the *bar-3* branch increases for $0.05\leq q^{\prime }\leq 0.7$ and then becomes constant before $q^{\prime }=0.3$, where a sudden jump occurs and the energy decreases. In Figure 8b, we plot $\Delta \omega^{\prime }$ for several values of ΔT*bar-1*. In contrast to the SB transitions, in which there is only one critical wave vector, for the TB transitions, we observe two critical ones: $q_{c_{1}}^{\prime }$ and $q_{c_{2}}^{\prime }$. We see a general trend in which $\Delta \omega^{\prime }$ is constant for $q^{\prime }\leq q_{c_{1}}^{\prime }$ and then suddenly decreases and becomes constant up to $q_{c_{2}}^{\prime }$. Then, it suddenly increases and becomes constant again. We notice that the $\Delta \omega^{\prime }$ value for $q^{\prime }\geq q_{c_{2}}^{\prime }$ is greater than that for $q^{\prime }\leq q_{c_{1}}^{\prime }$. Although each ΔT has the value of $\Delta \omega^{\prime }$ for $q^{\prime }\leq q_{c_{1}}^{\prime }$, for $q^{\prime }\geq q_{c_{1}}^{\prime }$, we see that higher values of $\Delta \omega^{\prime }$ correspond to higher values of ΔT. In addition, the range between the two critical points becomes shorter by decreasing ΔT.

**Figure 9:**
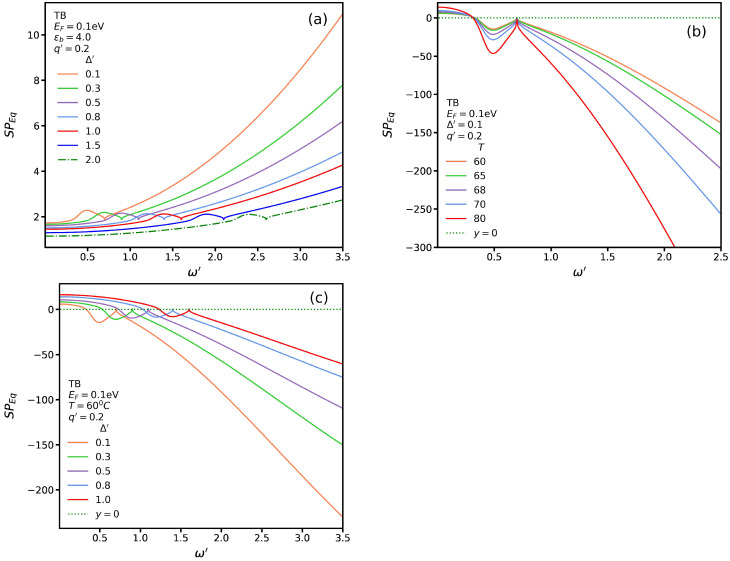
(**a**) SP*_Eq_* for TB transitions as a function of $\omega^{\prime }$ at several values of $\Delta^{\prime }$ for a constant permittivity: $\epsilon_{b}=4$ at $q^{\prime }=0.2$. (**b**) SP*_Eq_* for several temperatures at $\Delta^{\prime }=0.1$ eV. (**c**) SP*_Eq_* for several values of $\Delta^{\prime }$ at a constant temperature: *T* = 60 °C.

So far, we considered $\Delta^{\prime }=0$ in $\chi^{TB}$ in Equation (4). To study the influence of the induced energy gap in SP modes for TB transitions, in Figure 9a, we plot Equation (1) for several different values of $\Delta^{\prime }$ and a substrate with constant permittivities, such as $\epsilon_{b}=4$ with $q^{\prime }=0.2$, N=8, and $E_{F}=0.1$ eV. We note that there is no root for Equation (1). In other words, for substrates with constant permittivities, AGNRs do not support any TB SP modes. In Figure 9b, we plot Equation (1) for a VO_2_ substrate with $\Delta^{\prime }=0.1$ eV, $q^{\prime }=0.2$, and several temperatures. We observe that, similarly to the case in which $\Delta^{\prime }=0$, three SP modes exist. In Figure 9c, we plot Equation (1) for several values of $\Delta^{\prime }$ in an insulating phase in which T=0. For this typical temperature, we see that three roots exist. We note that the energy of the SP modes increases when the substrate-induced gap $\Delta^{\prime }$ is increased.

**Figure 10:**
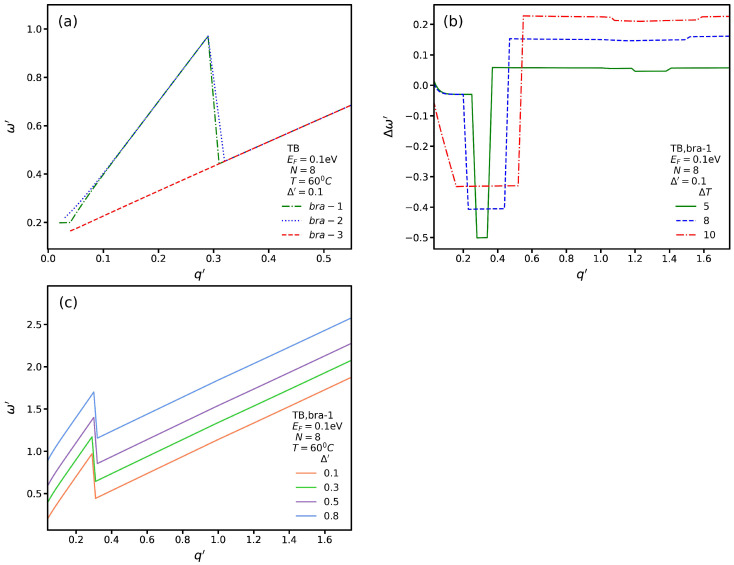
(**a**) SP spectrum for TB transitions for $\Delta^{\prime }=0.1$ eV and *T* = 60 °C. (**b**) $\Delta \omega^{\prime }$ as a function of $q^{\prime }$ for several values of ΔT for $\Delta^{\prime }=0.1$ eV. (**c**) The *bar-1* spectrum as a function of $q^{\prime }$ for several values of $\Delta^{\prime }$.

In Figure 10a, we plot the spectrum for three SP modes and TB transitions with $\Delta^{\prime }=0.1$ eV and *T* = 60 °C. We see that the group velocities of *bar-1* and *bar-2* are higher than that of *bar-3* for $q^{\prime }\leq 0.3$, while for $q^{\prime }>0.32$, all of the branches converge to one. In addition, we observe that for *bar-1* and *bar-2*, the SP mode energy is higher than that for *bar-3* and $q^{\prime }\leq 0.3$. In Figure 10b, we plot $\Delta \omega^{\prime }$ for several values of ΔT with $\Delta^{\prime }=0.1$ eV for *bar-1*. In contrast to Figure 8b, in which, for ΔT=10, the magnitude of $\Delta \omega^{\prime }$ is constant for $q^{\prime }\leq 0.2$, here, by increasing $q^{\prime }$, $\Delta \omega^{\prime }$ decreases and then reaches a constant value. In addition, similarly to Figure 8b, two critical wave vectors exist. In Figure 10c, we plot the SP spectrum of the first branch for different values of $\Delta^{\prime }$ at *T* = 60 °C. We observe a general trend in which the energy of the SP mode increases and then suddenly decreases, and later, it increases again with a lower group velocity. In addition, we note that by increasing the magnitude of $\Delta^{\prime }$, the energy of the SP modes increases.

## 4. Summary

We studied the effects of a VO_2_ substrate on the TM SP modes of metallic AGNRs within the Lindard approximation for both SB and TB transitions. We saw that for a vanishing induced gap $\Delta^{\prime }=0$, there are no SP modes for TB transitions, while there are for SB transitions. We noticed that the excitation energy of these modes in the insulating phase is smaller than that in the metallic phase. Moreover, the frequency of TM SP modes for SB transitions is smaller than that in suspended AGNRs characterized by $\epsilon_{1}=\epsilon_{2}=\epsilon_{0}$. The energy of the SP modes increases by moving from the insulating to the metallic phase of this phase-change material (PCM).

In contrast to the case in which a substrate with constant permittivity did not support TM SP modes for TB transitions, we noticed that AGNRs on a VO_2_ substrate support three TM SP modes. In addition, there was a degeneracy in the SP spectrum for a particular range of wave vectors. Then, we considered the case in which a substrate-induced gap existed and compared these results with those for a vanishing $\Delta^{\prime }$. We found out that for substrates with a constant permittivity, no TM SP modes existed for TB transitions, as in the case in which $\Delta^{\prime }=0$. However, for a finite $\Delta^{\prime }$, three TM SP modes existed, in which the energy increased with $\Delta^{\prime }$.

The obtained results indicate that PCMs, such as VO_2_, could be exploited to dynamically manipulate the state of plasmonic quantum-processor qubits based on AGNRs. For instance, changing the Fermi energy is the only available method for manipulating TM SP modes in AGNRs deposited on substrates that are in either a metallic or dielectric phase with a constant permittivity. Therefore, in this case, AGNRs can support only one TM SP photon at a time. However, as a PCM substrate whose permittivity can be changed with the temperature, VO_2_ provides the opportunity to have a substrate with a mix of metallic and dielectric phases. This results in the generation of two entangled photons at the same time within an AGNR qubit, which normally requires two qubits. In addition, the mode volume of TM SPs is significantly smaller in the presence of VO_2_ than in its absence. Consequently, SP modes exhibit a higher intensity. This leads to a nonlinear optical response with higher values, making it desirable to launch SP modes in graphene nanoribbons.

## Figures and Tables

**Table 1:** Values of the parameters in Equation (8).

*j*	$a_{j}$	$b_{j}$	$\hbar \omega_{j}$ (eV)
1	0.790	0.550	1.020
2	0.474	0.550	1.300
3	0.483	0.500	1.500
4	0.536	0.220	2.750
5	1.316	0.470	3.490
6	1.060	0.380	3.760
7	0.990	0.385	5.100

**Table 2:** Values of the parameters in Equation (9).

*j*	$c_{j}$	$d_{j}$	$\hbar \Omega_{j}$ (eV)
1	1.816	0.950	0.860
2	0.972	0.230	2.800
3	1.040	0.280	3.480
4	1.050	0.340	4.600

## Data Availability

Not applicable.
